# Nutrition and Breast Cancer: A Literature Review on Prevention, Treatment and Recurrence

**DOI:** 10.3390/nu11071514

**Published:** 2019-07-03

**Authors:** Paola De Cicco, Maria Valeria Catani, Valeria Gasperi, Matteo Sibilano, Maria Quaglietta, Isabella Savini

**Affiliations:** 1 Department of Pharmacy, School of Medicine, University of Naples Federico II, Via Montesano 49, 80131 Naples, Italy; 2 Department of Experimental Medicine, Tor Vergata University of Rome, Via Montpellier 1, 00133 Rome, Italy

**Keywords:** breast cancer, diet, nutrients, food, prevention

## Abstract

Breast cancer (BC) is the second most common cancer worldwide and the most commonly occurring malignancy in women. There is growing evidence that lifestyle factors, including diet, body weight and physical activity, may be associated with higher BC risk. However, the effect of dietary factors on BC recurrence and mortality is not clearly understood. Here, we provide an overview of the current evidence obtained from the PubMed databases in the last decade, assessing dietary patterns, as well as the consumption of specific food-stuffs/food-nutrients, in relation to BC incidence, recurrence and survival. Data from the published literature suggest that a healthy dietary pattern characterized by high intake of unrefined cereals, vegetables, fruit, nuts and olive oil, and a moderate/low consumption of saturated fatty acids and red meat, might improve overall survival after diagnosis of BC. BC patients undergoing chemotherapy and/or radiotherapy experience a variety of symptoms that worsen patient quality of life. Studies investigating nutritional interventions during BC treatment have shown that nutritional counselling and supplementation with some dietary constituents, such as EPA and/or DHA, might be useful in limiting drug-induced side effects, as well as in enhancing therapeutic efficacy. Therefore, nutritional intervention in BC patients may be considered an integral part of the multimodal therapeutic approach. However, further research utilizing dietary interventions in large clinical trials is required to definitively establish effective interventions in these patients, to improve long-term survival and quality of life.

## 1. Introduction

Breast cancer (BC) is the second most common cancer worldwide and the most commonly occurring malignancy in women (22.9% of female cancers), with more than 2 million of new cases diagnosed in 2018 [1,2]. Although the incidence is higher in Western Europe and North America, it is rising in developing countries, because of increased life expectancy, urbanization, and the adoption of western lifestyles [3]. According to the American Cancer Society, the five-year survival rate has improved from 63% in 1960 to 90% at present [4], thanks to earlier diagnosis with mammogram screening, and improved surgery and adjuvant treatment. Indeed, in 2018, BC death rates have rapidly slowed to 6.6% [5]. However, survivors are at increased risk of recurrence, even 20 years after the initial diagnosis [6]; in addition, they show increased risk to gain weight and develop other comorbidities, such as cardiovascular diseases or metabolic disorders [7,8,9].

**Figure 1:**
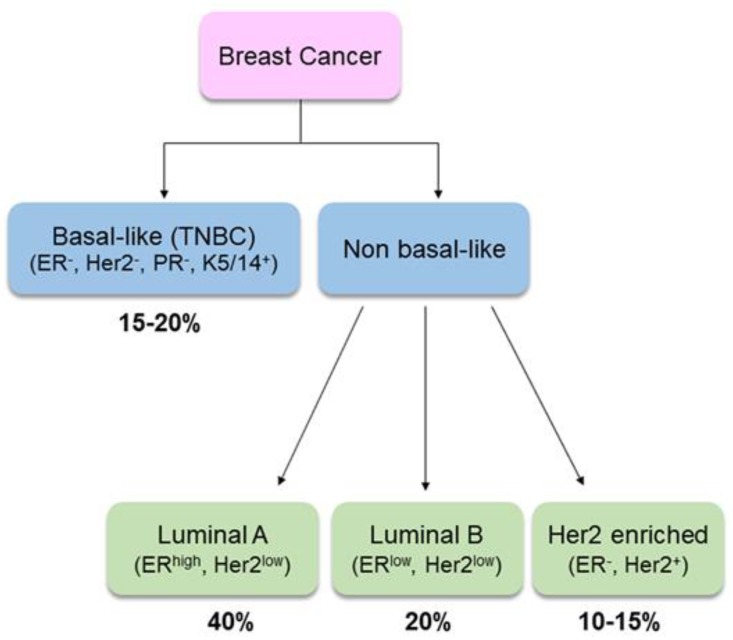
Breast cancer sub-types and relative prevalence. TNBC: triple negative breast cancer [12].

Clinically, BC is a heterogeneous disease. Gene-expression profiling has identified two main groups based on estrogen receptor (ER) expression: ER-expressing (ER^+^) breast tumors are more strongly associated with hormone-related factors than tumors that do not express it (ER^−^) [10]. According to cell types of origin (luminal or basal/myoepithelial cell compartment), BC is also classified as basal-like or NON-basal-like. The former, also known as “triple-negative”, accounts for about 10% of all BCs. It is characterized by the absence of all three hormonal receptors, i.e., ER, progesterone receptor (PR) and human growth factor-neu receptor (Her2), while it has high expression of basal cytokeratins. The non-basal like cancer can be further distinguished in luminal A (ER^high^/Her2^low^), luminal B (ER^low^/Her2^low^) or Her2-enriched (Figure 1). Due to the complexity of biology, understanding the etiological heterogeneity of BC subtypes will help in guiding treatment, predicting survival and informing prevention strategies [11].

Several risk factors have been identified: non-modifiable factors include older age (>65 versus <65 years), genetic predisposition (including DNA mutations and BC family history), early menarche (<12 years), late menopause (>55 years), age at first pregnancy over 30 years, infertility and not having children, use of contraceptives, hormonal treatment after menopause, and no history of breastfeeding [13,14]. Among modifiable lifestyle factors, dietary choices and being overweight or obese are associated with different risks of BC incidence and recurrence [15,16]; in particular, obesity is associated with poorer overall survival and increased mortality in post-menopausal BC women [17].

During recent decades, several studies have evaluated the relationship between specific foods (i.e. alcohol, fruits, vegetables, meat, soy food) and BC development. However, no consistent and statistically strong association has been established, except for alcohol intake [16]. Nonetheless, it has been proposed that diet may have a significant impact on BC outcomes. Consistent with dietary guidelines directed towards the general population, the adoption of a healthy dietary pattern, based on high consumption of fruits, vegetables, whole grains, poultry and fish, and low consumption of red meat, refined foods, sweets and high-fat dairy products, might improve the overall prognosis and survival of women diagnosed with early-stage BC (stage I, stage II, or stage IIIA) [18]. Moreover, a growing body of evidence strongly supports that physical activity is also associated with a greater chance of BC surviving [19].

Based on the most recent evidence, lifestyle recommendations were drawn up by the World Cancer Research Fund/American Institute for Cancer Research (WCRF/AICR) [20]. According to these recommendations, (1) maintaining a healthy body weight, (2) being physically active, (3) following a fiber- and soy-rich diet, and (4) limiting the intake of fats (in particular, saturated fatty acids) may improve overall survival after BC diagnosis [20]. Much evidence also supports the clinical relevance of nutritional intervention in patients with cancer, aimed at ensuring an adequate intake of energy and nutrients during chemotherapy, which may also result in improved response to and reduced toxicity of pharmacological anti-cancer therapies [21]. In addition, lifestyle changes including diet and exercise can reduce the long-term side effects of treatment protocols and promote long-term overall health by reducing BC comorbidities (e.g., obesity, hypertension, hyperlipidemia, and diabetes mellitus). Indeed, a potential new role for nutrition as armamentarium of the modern oncologic therapies is emerging.

Thus, the aim of this review is to provide an overview of current evidence on the relationship between nutritional factors and BC. In detail, we will highlight the influence of specific foodstuffs on the risk of BC incidence and recurrence. We will also summarize recent findings on the effect of diet during therapy, in order to gain a better understanding of the importance of nutritional intervention for cancer patient management.

## 2. Selection of Studies

A bibliographical search was performed in PubMed using combinations of key words relating to “breast cancer” AND “foodstuffs” (i.e. alcohol, fruits, vegetables, meat, soy food) OR “food nutrients” (i.e. dietary fiber, dietary carbohydrate, glycemic index, dietary fat and fatty acids) OR “fasting” AND “incidence” OR “survival” OR “recurrence” OR “mortality” OR “radiotherapy” OR “chemotherapy” OR “drug side effects”. The eligible criteria include studies in English language published between 2000 and 2019. We included studies referring to either breast cancer incidence, recurrence or survival. In particular, we focused on available meta-analyses and systematic reviews, large epidemiological studies, cohorts and case-control studies and randomized control trials. Information on clinical trials was from URL: http://clinicaltrials.gov/. Reference lists from selected articles were also manually checked to identify additional relevant reports. Titles and abstract were independently screened by author to determine study eligibility. Hence, we included articles that linked nutritional factors to (a) breast cancer incidence and recurrence; (b) disease-specific mortality or all-cause mortality; (c) breast cancer therapy (d) reduction of drug-related side effects. We considered only prospective cohort studies that had a total sample size of at least 200 subjects (with the only exception for studies relative to vitamin supplementation and drugs side effects). There was no restriction for the menopausal period or the cancer subtype or the type of anti-cancer therapy that patients received.

From the initial search, 361 papers including only humans were returned; among them, 207 articles derived from the combination “breast cancer” AND “foodstuffs” OR “food nutrients” AND “incidence” OR “survival” OR “recurrence” OR “mortality”, while 154 articles derived from the keywords “breast cancer” AND “foodstuffs” OR “food nutrients” AND “radiotherapy” OR “chemotherapy” OR “drug side effects”. After manual screening for duplication and available full-text articles, 249 articles were excluded and a total of 112 pertinent articles were selected for the specific scope of this review.

Additionally, global breast cancer facts and statistics were extracted from the web platforms of leading authorities (i.e. World Cancer Research Fund, International Cancer Societies and World Health Organization).

Finally, some in vitro and in vivo studies were also included to give more insight into the potential mechanism(s) of action underlying the effects observed in humans.

## 3. Dietary Factors in Breast Cancer Incidence and Recurrence

Adhering to a healthy lifestyle, including weight management and high-quality diet, influences both the risk of developing BC and post-diagnosis outcomes. Mainly, sedentary lifestyle and poor dietary habits, characterized by excessive intake of high-caloric foods (rich in sugar and saturated fats), as well as low intake of healthy foods (containing ω-3 fatty acids, natural antioxidants, fiber), ultimately lead to obesity. Such a condition contributes to increased adipose tissue inflammation, creating a favorable microenvironment for BC development and progression. Indeed, obesity is associated with both increased risk of post-menopausal BC and BC recurrence and mortality. A systematic literature review and meta-analysis of 82 follow-up studies, including 213,075 BC survivors and 41,477 deaths (23,182 deaths attributed to BC), showed a correlation between body mass index (BMI) and BC survival. In particular, an increased risk of 17%, 11% and 8% for overall mortality and 18%, 14% and 29% for BC-specific mortality has been observed for each 5 kg/m^2^ BMI increment (i) before BC diagnosis, (ii) less than 12 months after diagnosis and (iii) 12 or more months after diagnosis, respectively [22]. Besides BMI, some studies also reported a significant positive association between waist-hip ratio and BC mortality, in post-menopausal women [20,23]. 

Based on epidemiological and pre-clinical studies, some foods and nutrients (e.g., carbohydrates, saturated fat, red and processed meats) are considered potential risk factors for BC, as they increase circulating levels of endogenous estrogen, insulin-like growth factor (IGF)-1 and pro-inflammatory cytokines. In contrast, fiber, ω-3 poly unsaturated fatty acids (PUFAs), vitamins C and E, fruits and vegetables may have a protective role by reducing oxidative stress and lowering chronic inflammation (Table 1) [24].

Adhesion to the Mediterranean diet appears to be inversely linked to BC incidence and mortality, although evidence is still limited [20,42,43,44]. However, several case-control studies and randomized controlled trials have shown that the higher the adherence levels, the lower the BC incidence levels; indeed, poor diet, together with a sedentary lifestyle (e.g. physical inactivity), can increase the risk of developing BC both in Mediterranean Basin countries [45,46,47] and in countries displaying a tendency for “westernization in diet” [48,49].

### 3.1. Fruits and Vegetables

The heavy consumption of vegetables and fruits in the Mediterranean diet provides considerable amounts of both polyphenols and fiber, both of which have been suggested to prevent carcinogenesis [20,50,51,52].

A potential mechanism of action of polyphenols resides in their ability to counteract oxidative stress and inflammation. For example, polyphenols of blueberry powder can modulate BC proliferation and metastatic activity by regulating interleukin (IL)-6 [53]. Polyphenols are also able to inhibit the enzymatic activity of lipoxygenase (LOX) [54] and cyclooxygenase (COX) [55], as well as the activity of the transcription factor NF-κB [56]; these proteins can be overexpressed in tumor cells and, moreover, are important for regulating the expression of inflammatory cytokines, such as tumor necrosis factor α and IL-1 [57,58]. Finally, some polyphenols have been found to antagonize estrogen signaling, by either inhibiting aromatase, which is responsible for estrogen synthesis [59], or binding the ER receptor [60,61], thus regulating proliferation of tumor cells. Through a similar mechanism of action, fiber may prevent carcinogenesis by binding estrogens and reducing their serum levels or by improving insulin sensitivity and reducing weight gain [51].

Despite these findings, a large meta-analysis of 15 prospective studies found only a weak association between intake of fruits and vegetables combined, but not vegetables alone, with reduced BC risk [25]. The European Prospective Investigation into Cancer and Nutrition (EPIC) Italian study showed an inverse association between high consumption of leafy and fruiting vegetables, as well as raw tomatoes, and BC risk [27]. Accordingly, Fung et al. [26] examined the relationship between 29 different types of fruits and vegetables and risk of ER^−^ BC, among post-menopausal women; the authors found an inverse association between ER^−^ BC and intakes of blueberries, strawberries and peaches/nectarines. In contrast, no association of fruit, overall or by subtypes, with BC risk was found by Masala and collaborators [27]. In conclusion, according to the latest WCRF 2018 Report [20], evidence suggesting that the consumption of non-starchy vegetables decreases the risk of BC are limited, and no conclusions could be reached.

### 3.2. Red Meat

Red and processed meat represent risk factors for BC due to their heme iron content, administration of estrogens to cattle or mutagens created during cooking [62]. A recent comprehensive meta-analysis including 17 prospective studies evaluated the association of red and processed meat intake with BC risk: unprocessed red meat consumption was associated with a 6% higher BC risk, while processed meat consumption was associated with a 9% higher BC risk [28]. Further, a cohort study conducted in UK on 262,195 women demonstrated that processed meat consumption was associated with overall and post-menopausal (but not pre-menopausal) BC, whereas red meat consumption was not [29]. According to these results, cooking method, rather than red meat itself, represents a possible cause of increasing BC risk. High-temperature cooking increases the formation of potentially pro-carcinogenic compounds, including heterocyclic amines, N-nitroso compounds and poly-aromatic hydrocarbons [63]. Although there is no conclusive evidence, consistent with WCRF/AICR 2018, common recommendations are to not completely avoid eating meat (because it is a source of nutrients, like proteins, iron, zinc and vitamin B_12_), but instead, to limit consumption of red meat to no more than about three portions per week (equivalent to about 350–500 g cooked weight).

### 3.3. Dietary Fat

The role of dietary fat (and the type of consumed fat) on BC incidence has been studied in one of the largest randomized controlled trial conducted in the United States, the Women’s Health Initiative (WHI) Dietary Modification Trial. The study enrolled 48,835 post-menopausal women, subdivided in low-fat diet (20% of total energy) or usual dietary fat intake groups. The results indicate that a low-fat diet might reduce the risk of developing BC by approximately 9%, over an 8.1-year follow-up period; however, the estimated risk reduction was not statistically significant. Secondary analyses suggested a greater risk reduction among women who entered the trial having a habitual high-fat diet at baseline [30]. In addition, the type of dietary fat consumed, as well as the menopausal status, may also influence the risk. A meta-analysis study found higher BC risk in post-menopausal women consuming diets high in total fat and polyunsaturated fats. Conversely, dietary fat appears to have protective effects in pre-menopausal women [31]. A recent systematic review has reported that high saturated fat intake is associated with increased risk of BC-specific and all-cause mortality, whereas ω-3 fat intake is inversely associated with all-cause mortality [32]. Recently, the EPIC study, conducted in a large (*n* = 337,327) heterogeneous cohort of women, showed a positive association between high total and saturated fat intake and development of ER^+^/PR^+^ BC subtype, but not ER^−^/PR^−^ disease. The results of this prospective study indicate that a high-saturated-fat diet increases risk of BC and, most conspicuously, of receptor-positive cancer, particularly ER^+^ [33]. Biologically, dietary fats can influence the process of carcinogenesis by modulating intracellular signaling cascades [64]. Furthermore, accumulated adipose tissue may lead to metabolic syndrome and tumorigenesis, via pathways involving insulin and IGF-1 [65]. A recent meta-analysis indicated that dietary cholesterol is also associated with increased BC risk [34]. In conclusion, although high-fat diet, total cholesterol and triglyceride levels have mostly been found to be associated with increased risk, the evidence are limited.

### 3.4. Dairy Products

Dairy products contain a mixture of components (saturated fats, calcium, vitamin D, butyrate, lactoferrin and conjugated linoleic acid) that possibly influences BC risk in opposite directions; consistently, epidemiologic studies have produced contradictory results, reporting both inverse and positive associations. Differences in the outcomes of these investigations may be partly due to discrepancies in dairy consumption among different studies. No significant association between intakes of total dairy fluids or solids (per 100 g/die) and BC risk has been found in a meta-analysis of eight prospective cohort studies (from North America and Western Europe), including 351,041 women, of whom 7,379 were diagnosed with invasive BC during 15 years of follow-up [35]. Conversely, another meta-analysis of 18 prospective cohort studies, involving 24,187 cases and 1,063,471 participants, largely from the United States and Europe, indicated that increased consumption of total dairy food, but not milk, might be associated with reduced risk. As indicated by subgroup analyses, associations were more evident in pre-menopausal women and for low-fat dairy intake [36]. These findings have been confirmed by Zang et al. [37] in the biggest meta-analysis of 22 prospective cohort studies (1,566,940 participants) and five case-control studies (33,372 participants) extended to both Western and Asian population. In summary, they found a significant dose-, time-, and dairy-type-dependent relationship between dairy food consumption and BC development: therefore, it seemed that high (>600 g/day) and modest (400–600 g/day) dairy consumption more potently reduce BC risk, with respect to low dairy consumption (<400 g/day). Further subgroup analyses revealed that consumption of fermented dairy, yogurt or low-fat dairy products is inversely associated with BC development only in American women, after >10 years follow-up [37]. The protective effect can be explained with the anti-carcinogenic properties of several compounds present in dairy products. In particular, both in vitro and animal studies have shown that vitamin D inhibits BC development and that its dietary increase reduces experimental mammary tumor growth [66,67,68,69]. Accordingly, high intakes of calcium and vitamin D are moderately related to lower BC risk, particularly in pre-menopausal women [70]. However, dairy products also contain saturated fatty acids, endogenous IGF-1 (which has been shown to promote BC growth) and various contaminants, such as potentially carcinogenic pesticides, that might increase BC incidence. Indeed, high-fat dairy consumption may result in overall higher dietary fat intake, which can be pro-carcinogenic [31]. In contrast, low-fat dairy products lose the majority of their saturated fatty acids while retaining unsaturated fatty acids for which no significant association with BC risk has been demonstrated [71]. Thus, the heterogeneous composition of dairy product makes the net effect of dairy consumption on BC prevention difficult to settle.

### 3.5. Carbohydrate and Glycaemic Index

Available data about the association between intake of total carbohydrates, or specific types of carbohydrates (such as total sugars or specific sugars), glycemic index (GI) and glycemic load (GL) and BC risk are contradictory and inconclusive [72,73,74,75]. GI and GL are both measurements of carbohydrate quality. In particular, GI refers to the postprandial glucose response to a fixed amount of 50 g carbohydrates from different foods. GL is the product of GI and the total available carbohydrate content in a particular amount of food. Thus, GL is a stronger predictor of postprandial glycaemia and insulin response than GI.

In a recent meta-analysis [38], a weak increased BC risk (about 6%) has been reported to be associated with high GI in post-menopausal, but not in pre-menopausal women; for post-menopausal BC, the association was slightly stronger for women with hormone receptor-negative (ER^−^ and/or PR^−^) phenotype, but findings were not statistically significant. A majority of studies found that neither GL nor carbohydrate intake (range 112.3–343.5 g/day) are related to increased BC risk in pre- or post-menopausal women. However, after stratification by hormonal receptor status, the association becomes significant for women with ER^−^ and/or PR^−^ tumors; the same pattern has been observed in both pre- and post-menopausal women. No association between intake of total sugar or fructose and BC risk has been detected. Finally, the associations seem to be not modified by BMI.

The positive association between GL and ER^−^ BC risk in post-menopausal women might be due to increased insulin serum levels following carbohydrate consumption. In fact, insulin enhances growth hormone (GH) levels and, in turn, the synthesis of IGF-1 [76], which has mitogenic and antiapoptotic effects on BC cells [77,78]. However, even if there seems to be no association between carbohydrate intake, GI or GL and overall BC risk, glycemic control is advisable.

### 3.6. Alcohol

Alcohol consumption is the variable which is most consistently associated with BC onset and overall mortality. There is strong evidence that alcohol intake, regardless of the type of alcoholic drink consumed (beer, wine or spirits), and menopausal status are consistently associated with increased BC risk. In particular, a dose-response meta-analysis for pre-menopausal (*n* = 4,227 cases) and post-menopausal (*n* = 35,221 cases) women showed that for every 10 g ethanol consumed per day, there was an associated statistically significant increased risk of about 5 and 9%, respectively [20].

Such a positive association might relate to the ability of ethanol to promote epithelial-mesenchymal transition, tumor growth and metastasis formation [79,80]. Ethanol has also been shown to increase estrogen concentrations through several mechanisms: (i) increase of aromatase activity, (ii) inhibition of enzymes involved in estrogen degradation, (ii) decrease of melatonin secretion, which inhibits estrogen production and (iv) increase in hepatic oxidative stress that leads to inhibition of steroid metabolism. As a result, estrogens may exert their carcinogenic effect on breast tissue [81].

Heavy alcohol consumers usually show inadequate intake of many essential nutrients, including folate, which is crucial for DNA synthesis and repair, thus maintaining genomic stability. Alcohol is a well-known folate antagonist, thus reducing the bioavailability of the latter [82].

### 3.7. Soy Products and Isoflavones

Soyfoods are a dietary source of isoflavones, compounds with weak estrogen-like activity: their chemical structure is similar to endogenous human estrogens, with which they compete for binding to estrogen receptors. Three isoflavones, genistein, daidzein, and glycitein, are naturally present in the soybean itself and in most soy products, and account for approximately 50–55%, 40–45%, and 5–10% of total isoflavone content, respectively [83]. In recent years, the relationship between soyfoods and BC has become controversial because of concerns—based mostly on in vitro and rodent data—due to their oncogenic effects, i.e., mimicking the action of estrogens and stimulating cell proliferation in estrogen-sensitive breast tumors. However, soy constituents may possess anti-carcinogenic and anti-oxidant properties, as well as the ability to induce apoptosis and inhibit angiogenesis [84]. In addition, three meta-analyses showed that the consumption of soy isoflavones is inversely associated with BC incidence. This protective effect has only been observed among Asian populations, particularly in post-menopausal women, whereas no association has been found in Western populations. This finding could be due to the substantially higher consumption of soy food in Asian women (45.9 mg isoflavones/day) throughout their lifetime than Western women (3.2 mg isoflavones/day) [39,40,41].

## 4. Impact of Therapy on Nutritional Status of Women with BC

Many treatment options employed in BC therapy have been demonstrated to carry long-term toxicities. The therapeutic approaches include different chemotherapeutic agents, alone and/or in combination, as well as radiation, surgery (mastectomy or lumpectomy) or hormonal therapies, depending on the stage. Surgery and radiation therapy, often along with chemo or other drug therapies either before or after surgery, are commonly used to treat BC at stages I to III. Systemic therapy (chemotherapy, hormone therapy and antibody therapy) represents the standard treatment for stage IV BC and for distant recurrence. The most common chemotherapeutic regimes include CMF (cyclophosphamide, methotrexate, 5-flourouracil) or anthracyclines (epirubicin or doxorubicin) that have been demonstrated to reduce mortality by 35% [85]. Therapy usually runs 3-6 months and is often accompanied by side effects, including nausea, vomiting, loss of appetite, dry mouth and changes in taste or smell perception [86]. Weight gain is the most common side effect occurring in women receiving chemotherapy, and it is associated with a negative effect on quality of life and survival. As reported in the Women’s Healthy Eating and Living (WHEL), women treated with cytotoxic therapies have 65% increased risk of gaining weight during treatment, compared to women receiving other treatments, such as radiotherapy or hormonal therapy (tamoxifen or aromatase inhibitors) [87]. Increase in body weight after chemotherapy usually ranges between 1 to 5 kg, and may be associated with changes in body composition with increase in fat mass and loss in muscle mass, also known as sarcopenic obesity. Being overweight or obese during chemotherapy may negatively impact BC prognosis and overall survival, since it can influence other medical conditions, such as diabetes, heart disease, hypertension and hypercholesterolemia [88,89]. Weight gain normally occurs when energy intake exceeds energy expenditure. However, in BC patients receiving chemotherapy, caloric intake usually decreases over the first year after diagnosis; therefore, weight gain may not result from overeating, but rather, may be related to lower physical activity and reduced resting metabolic rate. A 50% reduction in activity level can be observed in women subjected to chemotherapy, surgery and radiation, because of the constant fatigue or lack of energy. In addition, chemotherapy often impairs glucose metabolism and induces premature menopause that may influence weight gain and tumor growth pathways in BC patients [88,90]. The strongest evidence that weight loss resulting from physical activity is associated with better outcomes for BC patients comes from a big-pooled analysis, the After Breast Cancer Pooling Project (AFCPP), evaluating the post-diagnosis lifestyle factors and outcomes in four prospective cohorts of BC survivors. The study project reported 27% decreased risk of mortality in women who performed at least 10 Metabolic Equivalent per Task (MET)-hours per week, corresponding to 3–5 hours walking/week [91]. Moreover, cohort analyses and small randomized trials have shown that lifestyle interventions (specific dietary patterns or increased physical activity) significantly reduce secretion of insulin, estrogens, IGF-1 and inflammatory markers [92]. Thus, maintaining a healthy weight in BC women, by increasing physical activity and decreasing body fat, may be a reasonable intervention to improve prognosis.

Finally, it should be underlined that low BMI (<18.5 kg/m^2^) is also associated with poorer prognosis. Indeed, therapy-induced nausea has a substantial impact on eating enjoyment, leading to inadequate energy and essential nutrient intakes, and resulting in malnutrition, reduced compliance with treatment regimens, reduced immunity, emotional distress and negative quality of life [93,94,95]. Although this phenomenon appears to be possibly related to major vulnerability of underweight women to treatment [89], fortunately, these effects are transient, and recover after the end of chemotherapy.

## 5. Nutritional Interventions during BC Treatment

Changes in taste during BC treatment are mainly due to damage to taste receptor cells (TRCs) localized on the tongue epithelium and throughout the digestive tract caused by radiation or chemotherapeutic agents. Xerostomia (dry mouth) has also been implicated in taste change, as radiation therapy frequently affects saliva quantity and composition by damaging salivary glands. During chemotherapy, women report altered food preferences for macronutrients, which results in significant lower intake of proteins and fats [95]. An appropriate nutritional counselling can guide patients to adopt appropriate strategies in order to increase food palatability. For example, adding artificial flavors, eating smaller and more frequent meals, using more condiments, adding something sweet to meats, eating more boiled foods, eating candy before meals, drinking sweetened drinks, using plastic eating utensils, drinking from a straw or cooking in non-metal pots and pans can help to reduce the metallic taste frequently associated with meat. Lemon juice, chewing gum and mint also make meals more pleasant. Moreover, patients should maintain good oral hygiene by brushing their teeth and tongue before meals and using baking soda and salt wash or antibacterial mouthwash, as these may also contribute to changes in taste [96].

Some chemotherapeutic drugs may cause chelation of zinc and other heavy metals, leading to zinc depletion and contributing to loss of taste. Several clinical trials demonstrated that zinc supplementation might be useful for patients undergoing cancer chemotherapy in improving taste perception. Another valuable aid in reducing taste alteration is represented by amifostine, an organic thiophosphate that antagonizes damage of salivary glands triggered by radiation [97]. Some foods, including creams prepared with unrefined rice, selected cooked vegetables and vegetable and miso (an essential aminoacid-enriched condiment traditionally added to foods) soups, can prevent gastrointestinal symptoms appearing during chemotherapy [98]. Cereal creams, for example, avoid the irritating effect on the gut mucosa of a large amount of fibers and, in parallel, provide the nutritional advantage of whole grain cereals, while animal protein intake is usually reduced to prevent acidosis.

Beside limiting drug-induced side effects, some dietary constituents can also enhance therapeutic efficacy, thus improving the quality of life for cancer survivors. In the next paragraphs, we will describe some of the most relevant studies about the effects of specific nutrients on cancer therapy (Table 2).

### 5.1. ω-3 Poly Unsaturated Fatty Acids (PUFAs)

Eicosapentaenoic (EPA) and docosahexaenoic (DHA) acids are ω-3 PUFAs which are naturally found in marine organisms, whose intake has been reported to reduce BC incidence, in a dose-dependent manner (5% lower risk for each 0.1 g/day increment) [111]. In vitro and in vivo studies also demonstrated that ω-3 PUFA induce chemosensitization, maybe resulting from selective cytotoxicity on cancer cells without any effect on normal cells, *via* multiple pathways. One hypothesized mechanism resides in their chemical structure: ω-3 PUFAs are unsaturated and highly peroxidizable fatty acids rapidly incorporated into membrane phospholipids and lipid rafts of BC tumor cells. Consequently, membrane integrity is impaired and causes alteration or sequestration of membrane proteins (e.g., adapter proteins, receptor-associated enzymes, protein kinases and phosphatases) involved in survival and death. It has been demonstrated that ω-3 PUFAs tend to accumulate specifically in tumor cell membranes that are deficient in PUFAs [112].

Another well-established mechanism is generation of lethal levels of reactive oxygen species (ROS) and inhibition of anti-oxidant activities in cancer cells. Both mechanisms have important therapeutic potential resulting in improvement of efficacy of conventional anticancer therapies, especially against tumors otherwise resistant to treatments. EPA and DHA can also produce inflammation resolving metabolites; in particular, EPA is a substrate of COXs and LOXs for the synthesis of prostaglandins, thromboxanes and leukotrienes with anti-inflammatory and anti-tumorigenic properties, thereby inhibiting tumor cell growth and invasion. Conversely, it has been suggested that membrane enrichment with EPA and DHA leads to enhanced generation of resolvins and protectins, thus protecting normal cells against toxic chemicals, including chemotherapic drugs [113]. Finally, ω-3 PUFAs can bind nuclear receptors in tumor cells, modulating the expression of target genes involved in lipid metabolism and cell death [114]. These pleiotropic and multifaceted effects have led ω-3 PUFAs being tested as potential adjuvant of traditional chemotherapies. A small phase II trial, enrolling 25 metastatic BC patients (pre- and post-menopausal; PR/ER positive and negative) treated with anthracycline-based chemotherapy, has underlined safety and potential benefits of DHA supplementation (1.8 g/day). Data from this study reported increased disease-free survival in the sub-population of patients with high DHA incorporation into plasma phospholipids; furthermore, a slightly lower toxicity of chemotherapy, regarding anemia, thrombopenia and gastrointestinal toxicity, has also been observed [99]. A phase III trial, assessing the effects of ω-3 fats on chemotherapy efficacy and toxicity in 65 patients with metastatic BC (DHALYA NCT01548534), has been completed [115], further supporting the finding that DHA may specifically chemosensitize tumors to chemotherapy.

In addition, EPA and DHA are safe (absence of cardiotoxic effects) and effective in reducing the common chemotherapy-related side effects, such as bone density loss, peripheral neuropathy and weight gain. Loss of bone density and increased fracture rate are a side effect of cytotoxic chemotherapy in pre-menopausal women or of aromatase inhibitors (AI) in post-menopausal women. A small randomized pilot trial suggests that 4 g/day EPA plus DHA inhibits bone reabsorption in post-menopausal BC survivors receiving AI therapy [100]. A recent study demonstrated that ω-3 PUFAs supplement, in post-menopausal obese BC patients treated with AI, significantly reduces AI-associated arthralgia [101]. Cognitive and neuronal side effects are also commonly observed in women undergoing chemotherapy. Cancer patients experience impairments in attention, processing speed, executive function and working memory, with several grade of gravity depending on agents used, intensity and duration of treatment, and predisposing factors [116]. Interestingly, in a small-randomized trial, carried out on 20 BC patients receiving paclitaxel therapy, EPA (0.19 g/day) and DHA (1.04 g/day) oral supplementation reduces incidence of neuropathy from 60 to 30% [102]. However, a diet rich in added sugars (sucrose and fructose) induces a reduction of ω-3 PUFA neuroprotective activity, resulting in increased neuroinflammation, reduced neurogenesis and cognitive deficits [117]. Finally, by combining DHA supplementation with dietary energy restriction, BC-related obesity can be counteracted, for the dual effect on fatty acid metabolism and cell growth pathways, leading to inhibition of cell proliferation and induction of apoptosis; however, the effects in humans have to be proven [118]. Thus, for improvement of patient quality of life, it is possible that EPA and/or DHA will soon be recommended as adjuvants for chemotherapy and radiotherapy in BC patients.

### 5.2. Green Tea

Green tea is made from the steamed and dried leaves of *Camellia sinensis*, an evergreen shrub native to East Asia, the Indian Subcontinent and Southeast Asia. Green tea contains catechins, a large group of flavonoids, polyphenolic compounds with antioxidants properties. Epigallocatechin3-gallate (EGCG), the main representative catechin, shows strong chemopreventive and chemotherapeutic effects against BC. Indeed, experimental trials suggest a synergic and additive effect of EGCG with conventional cancer therapies, as well as amelioration of related side effects thanks to its anti-inflammatory and antioxidant activities [119,120]. In vitro and animal studies demonstrated that EGCG exhibits antiproliferative and pro-apoptic activities in tumor cells, through the inhibition of multiple signaling pathways, including COX-2 overexpression and activation of NF-κB-, MAPK-, IGF-1- and epidermal growth factor (EGF)-mediated signal transduction pathways. Additionally, EGCG can inhibit angiogenesis and tumor invasiveness, as well as modulate the immune system function [121]. Combination of green tea catechins with tamoxifen or paclitaxel appears an appealing strategy to enhance treatment of both ER^+^ and ER^−^ BC, while ameliorating the chemoterapic safety profile [120]. Two Japanese observational studies showed that consumption of a minimum of 5 cups of green tea in BC patients is associated with decreased risk of recurrence, particularly in those with early stage (I and II) disease, whereas no improvement has been observed for stage III patients [103,104]. Recently, a population-based prospective cohort study of 5042 BC patients in Shanghai, China (the Shanghai Breast Cancer Survival Study) demonstrated that tea intake (about 100 g of dried tea leaves/month) during the first 60 months after cancer diagnosis is associated with improved survival among women with triple-negative BC [105]. In addition, due to their ROS-scavenging properties, green tea polyphenols can circumvent the adverse effects induced by chemotherapeutic agents; in particular, preclinical studies have shown the beneficial effect of EGCG in reducing cardiac damage resulting from treatment with doxorubicin [122]. Green tea has been consumed safely for thousands of years without restriction for eventual toxicity. However, the clinical trial evidence is insufficient to make recommendations for the use of green tea as BC adjuvant treatment.

### 5.3. Antioxidants Vitamins and Minerals

The use of multivitamin (vitamins C, E and D) and mineral (selenium and calcium) supplements in cancer patients is very popular, because of their potential anticancer properties; they may also reduce oxidative damage triggered by chemo- and radio-therapy [123,124]. Observational data from the LACE study showed that 72% of BC patients were self-prescribing multivitamins; however, neither benefıcial nor harmful effects of these supplements have been observed. Multivitamin use, along with diet rich in fruits and vegetables and physical activity, may be beneficial in improving BC outcomes [125]. The controversial discussion regarding supplementation with antioxidant agents during cancer treatment is mainly due to their potential interaction with conventional cancer treatments. Since radiotherapy and many chemotherapic drugs (e.g., anthracyclines) exert their anticancer effects through ROS production, antioxidant agents may reduce their efficacy protecting both normal and tumor cells from oxidative damage [126]. Based on accumulating evidence from phase II and III trials, the widespread use of antioxidants during chemo and radiation treatments is not convincing.

Vitamin C, or ascorbic acid, is a water-soluble vitamin involved in several biological processes, including biosynthesis of collagen, neurotransmitters and L-carnitine, iron absorption and immune functions. As shown by in vitro studies, vitamin C can induce apoptosis of cancer cells and enhance immune response [127,128]. However, the effects of vitamin C supplementation on BC mortality or recurrence are controversial and seem to be dependent on dose, source of vitamin C, route of administration (oral versus intravenous) and timing and duration of supplementation [129,130]. The association between vitamin C (500 mg daily) and vitamin E (400 mg daily) supplementation during chemotherapy with tamoxifen, in post-menopausal women with BC, has been demonstrated to preserve from lipid peroxidation and DNA damage, restoring an adequate antioxidant state [106,107]. Similar findings have been obtained in studies on intravenous vitamin C administration [131,132]. Before high-dose vitamin C infusion, glucose-6-phosphate dehydrogenase deficiency should be ruled out, as red blood cell hemolysis may occur in people found to be deficient in the enzyme. There is also a concern on renal calculi in oxalate kidney stone formers. History of renal calculi should be established, and patient serum creatinine and renal function should be monitored regularly during treatment.

Vitamin E is a group of eight liposoluble vitamins—comprising four tocopherols and four tocotrienols—with antioxidant and anti-inflammatory properties. Vitamin E-rich foods include nuts, seeds, vegetable oils, green leafy vegetables and fortified cereals. Beside the beneficial effects observed with co-administration of vitamin C and E, other studies have shown that long-term vitamin E uptake can have negative effects [133]. High alpha-tocopherol levels (obtained as dietary supplements with amounts of more than 300 mg/day) may lead to interactions with tamoxifen, resulting in decreased antiproliferative activity [108]. On the contrary, the synthetic derivative alpha-tocopheryl succinate improves cell sensitivity to doxorubicin [134].

Selenium is an antioxidant mineral which is crucial for the activity of antioxidant enzymes (e.g., glutathione peroxidase) that participate in the metabolism of oxidants and drugs. In human whole blood, physiological selenium concentrations should be between 120 and 140 μg/L, depending on adequate selenium intake from dietary sources (e.g., grains, cereals, organ meats and seafood, with lower amounts in dairy products, fruits, and vegetables). Usually, patients with BC have significantly lower whole blood and serum selenium levels [135]. Selenium supplements are available as organic form of selenomethionine or inorganic form of sodium selenite, normally preferred in complementary cancer therapy. Selenium supplementation seems to reduce the side effects of conventional cytotoxic therapies (e.g. nephrotoxicity by cisplatin, mucositis by radiotherapy) without affecting their antitumor efficacy, thereby guarantying better compliance, fewer therapy dropouts and higher possible dosages. However, selenium is toxic if taken in excess and can lead to selenosis with gastrointestinal disorders, hair loss, sloughing of nails, fatigue, irritability and neurological damage. Thus, supplementation in cancer patients should be preceded by evaluation of selenium blood levels to avoid overdosing and side effects [136].

Vitamin D is a liposoluble vitamin mainly obtained through endogenous synthesis via sun exposure of the skin and, minimally, from dietary sources (oily fish, cheese and fortified foods, such as cereal, milk and dairy products, beef, and liver). When necessary, vitamin D can be taken up as supplement in the form of ergocalciferol (vitamin D_2_) or cholecalciferol (vitamin D_3_). Both forms need to be metabolized via hydroxylation, in liver and kidney, to the active metabolite 1,25-(OH)_2_ vitamin D_3_ (calcitriol). Physiologically, vitamin D has an essential role in skeletal mineralization, as it regulates intestinal calcium absorption, as well as bone and renal calcium reabsorption, thus contributing to maintenance of calcium and phosphorous plasmatic concentrations [137]. In cancer, vitamin D has been demonstrated to regulate the expression of genes involved in cancer development and progression, stimulating cell differentiation and apoptosis or inhibiting cell proliferation, angiogenesis, invasion, inflammation, and metastatic potential [138]. As shown by clinical and epidemiological studies, vitamin D deficiency is common among BC patients and it is considered a negative prognostic factor [139,140,141,142,143,144,145,146,147]. The biological effects of the active vitamin D metabolite calcitriol are mediated by binding to the vitamin D receptor (VDR); specific polymorphisms of the *vdr* gene have been associated with BC risk, since they may influence individual responsiveness to vitamin D among patients with cancer [148]. Vitamin D represents an effective approach for reducing development of osteoporosis in patients treated with aromatase inhibitor-based therapy [109]; vitamin D supplementation (50,000 IU per week) is indeed able to lessen joint pain and fatigue associated with letrozole treatment (an aromatase inhibitor) [110]. In BC patients, whose bone density can be affected by chemotherapy-induced menopause and aromatase inhibitors, clinical practice guidelines recommend supplementation with vitamin D and calcium, since vitamin D supplementation alone has reported no benefits for bone density or fracture risk [149].

Calcium is the most prevalent mineral in the body; it has been positively related to BC aggressiveness in pre-menopausal women with or without overweight [137,150]. Recommended doses range between 10 and 25 μg vitamin D and 1000 to 1500 mg calcium [149]. In this context, it should be underlined that calcium supplementation has been linked to increased risk of cardiovascular diseases [151]; therefore, future randomized trials are needed to assess safety of supplementation in BC patients undergoing chemotherapy.

### 5.4. Intermittent Fasting

Some regimens of fasting or caloric restriction may protect animals and cancer patients from toxic effects of oxidative stress and chemotherapeutic agents, and sensitize cancer cells to chemotherapy, a phenomenon known as differential stress resistance. Indeed, in healthy cells, nutrient deprivation results in inhibition of insulin/IGF-1 and phosphoinositide 3-kinases/protein kinase B pathways promoting cell growth, in order to invest energy in maintenance and DNA repair pathways that contribute to resistance to chemotherapy. In contrast, tumor cells are unable to activate this protective response, due to uncontrolled activation of growth pathways by oncogenic mutations [152]. Interestingly, a preliminary report on a small and heterogeneous group of 10 cancer patients demonstrated that short-term fasting pre- and post-chemotherapy is well-tolerated and associated with reduction in multiple chemotherapy-induced side effects (such as fatigue, weakness and gastrointestinal symptoms), without causing long-term weight loss; in cases where cancer progression can be assessed, fasting does not reduce the efficacy of chemotherapy [153]. Recently, a randomized-controlled pilot trial has been conducted on 13 women with Her2^−^, stage II or III BC, undergoing neoadjuvant treatment with TAC (docetaxel, doxorubicin and cyclophosphamide). Seven of them were fasted 24 hours before and after chemotherapy infusion, while the remaining ate normally: subjects in the fasting group were only allowed to drink water and coffee or tea without sugar. At the end of the scheduled treatment (6 cycles), no significant differences have been found, in occurrence of chemotherapy-related adjustments, between the two groups. However, in the fasting women, reduced hematological toxicity has been found, with significantly higher erythrocyte and thrombocyte counts even 7 days after chemotherapy [154]. No serious adverse effects have been observed in patients fasting up to 72 hours. However, larger randomized trials, such as the DIRECT study (NCT02126449), are now ongoing to evaluate the effect of fasting on tolerance and efficacy of neoadjuvant chemotherapy in women with stage II or III BC.

## 6. Nutritional Interventions to Reduce BC Recurrence and Mortality

Several dietary intervention trials have been conducted in BC patients during chemotherapy to improve health outcomes. The two bigger studies were the Women’s Intervention Nutrition Study (WINS) and the WHEL study. The first one was conducted on 2437 post-menopausal women with stage I or II BC receiving standard cancer management. During this trial, the hypothesis that dietary fat reduction improves relapse-free survival rate was tested. In the intervention group, fat intake was reduced from 29.2% to 20.3% of total calories, while maintaining nutritional adequacy. After a median follow-up of 5 years, relapse-free survival was 24% higher in the intervention group than in the normal diet group (30% of total energy from fat). Additionally, the relapse-free survival rate was greater in women with ER^−^ or/and PR^−^ disease than in women with receptor-positive disease. Further, significant reduction in body weight of approximately 6 pounds has been observed. However, reducing fat (fats, oils and sweets) necessarily led to make healthier choices; in fact, the percentage of subjects consuming fruit and vegetable increased. Thus, changes in intake of other nutrients besides fats in the intervention group might influenced the risk of BC recurrence [155]. The second randomized controlled study, the WHEL study, examined a different dietary intervention, in 3080 pre- and post-menopausal patients with early-stage disease. Dietary intervention consisted of increased vegetable servings (five servings/day and 16 oz of vegetable juice), fruit (three servings/day) and fiber (30 g/day) intake and reduced fat intake (15–20% of total calories). BC survivors were counselled with home telephone and cooking classes to support adherence to post-diagnosis diet. In addition, the control group received advice to eat at least five portions of fruit and vegetables each day (five-a-day advice). After 7.3-year follow-up, no evidence that the adoption of a dietary pattern high in vegetables, fruit and fiber and low in fat prevents BC recurrence or death has been observed [156]. Recently, a prospective study, the Cancer Prevention Study-II Nutrition Cohort (CPS-II Nutrition Cohort), conducted among 4452 BC survivors, evaluated whether pre- or post-diagnostic dietary intake consistent with the American Cancer Society (ACS) recommendations for cancer prevention were associated with BC mortality. While no associations between fruit and vegetable or whole grains intake and BC survival were found, an inverse association was observed with red and processed meat consumption and overall mortality [157]. In the Life After Cancer Epidemiology (LACE) study, the association among post-diagnosis dairy intake and increased overall mortality among women diagnosed with early-stage invasive BC was evaluated. In first analysis, no statistically significant relation has been found; however, in a second sub-analysis, high-fat dairy intake showed positive correlation with overall mortality and BC-specific mortality. These findings were consistent with the hypothesis that dairy fat intake may increase estrogen levels [158].

The consumption of dietary fiber in BC survivors and its relationship to prognosis has recently been investigated. In the Health, Eating, Activity, and Lifestyle (HEAL) study (*n* = 1183 survivors), fiber intake of >8.8 g/day results inversely associated with BC-specific and overall mortality [159]. Consistent with these findings, in a separate cohort study (*n* = 516 survivors) an inverse association between dietary fiber intake and overall mortality has also been observed [160]. The Nurses’ Health Study (*n* = 3846) reported decreased risk of overall mortality, after initial BC diagnosis, only for cereal fibers [161], whereas the WHEL trial found no relationship between high fiber intake and BC events or mortality [156]. Overall, evidence suggests that dietary fiber intake (at least 10 g/day, approximately equivalent to three slices of whole grain bread) significantly decreases risk (about 12%) of all-cause mortality [18].

Soy food consumption in BC survivors has raised concern about its safety due to the anticancer, but also estrogen-like properties of isoflavones. Epidemiologic data about post-diagnosis soy intake and BC outcomes are insufficient. To date, several studies indicate that soy food intake is inversely associated with mortality and recurrence in Chinese BC women, whereas the evidence is still limited for Western women, for whom soy product consumption is much lower [41,162,163]. A recent pooled analysis on 9514 BC survivors from both US and China showed no significant association between post-diagnosis soy food intake (10 mg isoflavones/day) and reduced risk of all-cause and BC-specific mortality, whereas statistically significant association with reduced recurrence risk has been observed [164]. Consistent with these findings, a multi-ethnic cohort study of women diagnosed with BC living in North America (17% Hispanics, 12% Blacks, 11% Asian Americans) found a significant trend of lower all-cause mortality associated with higher dietary intake of isoflavones (>10 mg/day). This association has similarly been seen across all racial/ethnic groups, but only in women with negative tumor hormone receptors (ER^−^, PR^−^) or those not receiving hormonal therapy [165]. Thus, even if the evidence that post-diagnosis consumption of soy-containing foods reduces the risk of all-cause mortality is limited, it can be considered safe in all women with BC, regardless of hormonal status. In conclusion, a daily reasonable amount of whole soy foods (about 30g, which provides 10-20 mg of soy isoflavones) is potentially beneficial for women with BC, while supplemental soy protein and isoflavone isolates should be avoided.

## 7. Conclusions

In the last twenty years, the concept of nutritional support as part of a comprehensive cancer management program has gained increasing interest.

**Figure 2:**
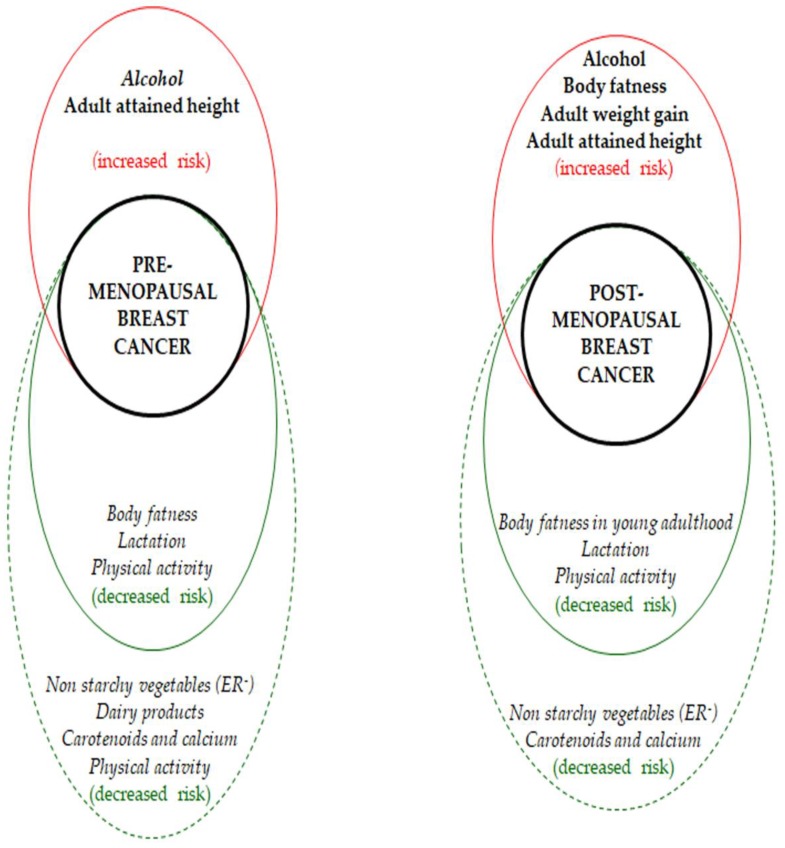
Main findings on breast cancer risk [19]. Red circle: direct correlation. Green circle: inverse correlation. Strong evidence: continuous line. Limited, but suggestive, evidence: dotted line. Convincing evidence: bold. Probable evidence: italic.

Only limited evidence exists for an association between main food groups and BC risk (as stated by the WCRF 2018 report) (Figure 2) [20]. The convincing evidence that adult body fatness and body fatness in young adulthood lower the risk of pre- and post-menopausal breast cancer, respectively, is, however, not taken into account when making recommendations. Being overweight or obese is, indeed, associated with increased risk of developing certain forms of cancer and, for some of them, the increase in risk is found with increasing body fatness even within the “healthy” range [20]. For this reason, the Expert Panel of WCFR recommends avoiding weight gain in adult life [20].

Some evidence suggests nutritional intervention as a key factor in determining cancer prognosis, patient quality of life and, notably, efficacy of anti-tumor therapies. Among BC patients, diet, physical activity and weight management, indeed, play a major role in improving survival. Patients with BC are often either overweight or obese at diagnosis and obesity is associated with increased overall and BC-specific mortality. Moreover, even without weight gain, women are affected by adverse changes in body composition, with frequent sarcopenia, accompanied by fat gain, which represents a significant risk factor for development of comorbidities (like cardiovascular diseases and diabetes), thus influencing long-term survival. Therefore, in these patients, nutritional intervention should be considered an integral part of the multimodal therapeutic approach, to reduce the risk of recurrence, mortality, and development of other chronic diseases.

Current evidence proposes that greater adherence of BC survivors to the Mediterranean diet pattern may reduce (i) BC recurrence, (ii) overall cancer mortality and (iii) other comorbidities, including cardiovascular diseases, with beneficial effects on health and longevity [42,43,44]. The mediterranean diet represents a significant source of bioactive compounds that could explain, at least in part, the beneficial effect on BC. Among the various mechanisms proposed, there are: reduction of body weight and waist circumference, amelioration of patient biochemical profile with lowering of glucose and insulin blood levels and increasing of antioxidant capacity. Therefore, the latest guidelines for nutritional treatment for cancer survivors, developed by the European Society for Clinical Nutrition and Metabolism (ESPEN), recommend a healthy dietary pattern characterized by high intake of vegetables, fruits and whole grains, low-moderate intake of dairy products, limited intake of red meat (no more than about three portions per week), and very little, if any, processed meat, as well as of sugar, sweets and alcohol.

BC patient quality of life is negatively impacted by treatment-related toxicity that also limits the enjoyment of eating. However, toxicity often remains an underestimated issue in the management of patients with cancer. Women with BC history often recur to nutritional supplements (like multivitamins and antioxidants), for management of typical symptoms and adverse effects of conventional cancer therapy. However, these products can have either positive (e.g., synergic effects) or negative (e.g., metabolic and drug interactions, diminishing the therapeutic benefits of conventional cancer treatments) effects and more clinical studies concerning safety and efficacy, as well as timing and dosing, are necessary. Until otherwise noted, the best advice given by the ACS is to get vitamins, minerals and antioxidants through food sources rather than supplements.

Despite the number of studies, nutrition science usually shows imprecise and/or contradictory results. Non-communicable diseases (like cancer, diabetes, obesity and heart disease) are multifactorial illnesses, and diet, although related, is only one of the risk factors (together with lifestyle choices, genetics and environmental factors) accounting for the pathology. In addition, in nutritional research, several limitations can be encountered that can cause confusion when interpreting of results. First, clinical trials can be useful for addressing simple and short-term questions, but not for studying long-term illnesses: it is very hard to randomly assign different diets to different groups of people and follow them for many years in order to establish whether a certain food is linked to a certain non-communicable disease. Observational studies give rise to more valuable study designs, but they are not controlled and, therefore, the presence of confounding factors make these investigations less precise. Other concerns relate to food surveys (often inaccurate and non-realistic), individual response to food components (different subjects have different responses to the same food, due to influences like gene-nutrient interactions, gut microbiota composition, etc.) and food nutrition profiles (influenced by food manipulation and storage: fresh vegetables are chemically different from processed vegetables). Despite these limitations, suggestive conclusions can be drawn if different types of studies (with different settings, methodologies and enrolled subjects) all point in the same direction; altogether, they may give a quite good indication about the link between a specific food component and a definite health outcome.

Based on these findings, it would be better to simply suggest a “healthy” dietary pattern, rather than making claims about the effects of specific foodstuffs or food components, and BC patients should be encouraged to improve their lifestyle and dietary habits before, during and after treatment, in order to have better long-term survival and quality of life.

## Figures and Tables

**Table 1:** Possible effects of dietary factors on BC risk.

	Study	Results	Reference
Fruits, vegetables	Meta-analysis (15 prospective studies)	RR = 0.89 (95% CI, 0.80–0.99, *p* = 0.67) fruits + vegetables; highest *vs.* lowest intake RR = 0.92 (95% CI, 0.86–0.98, *p* = 0.36) fruits; highest *vs.* lowest intake RR = 0.99 (95% CI, 0.92–1.06, *p* = 0.26) vegetables; highest *vs.* lowest intake	[25]
	Prospective study (75,929 women, 38–63 years, 24 years follow-up)	RR = 0.82 (95% CI, 0.71–0.96, *p* = 0.01), 2 servings/week of total berries RR = 0.69 (95% CI, 0.50–0.95, *p* = 0.02), 1 serving/week of blueberries RR = 0.59 (95% CI, 0.37–0.93, *p* = 0.02), 2 servings/week of peaches/nectarines	[26]
	Prospective study (31,000 women, 36–64 years, 11.25 years follow-up)	HR = 0.70 (95% CI, 0.57–0.86, *p* = 0.0001) leafy vegetables, highest *vs.* lowest quintile HR = 0.75 (95% CI, 0.60–0.94, *p* = 0.01) fruiting vegetables, highest *vs* lowest quintile no association with fruit	[27]
Red meat	Meta-analysis (13 cohort, 3 case-control, 2 clinical trials)	RR = 1.06 (95%CI, 0.99–1.14) unprocessed red meat, highest *vs.* lowest intake RR = 1.09 (95%CI, 1.03–1.16) processed red meat, highest *vs.* lowest intake	[28]
	Cohort study (262,195 women, 7 years follow-up) Meta-analysis	HR = 1.21 (95% CI, 1.08–1.35, *p* = 0.001), >9 g/day processed red meat RR = 1.09 (95% CI 1.03–1.15, *p* = 0.662), >9 g/day processed red meat in post-menopausal women RR = 0.99 (95% CI 0.88–1.10, *p* = 0.570), >9 g/day processed red meat in pre-menopausal women	[29]
Dietary Fat	Randomized controlled trial (48,835 post-menopausal women, 8.1 years follow-up)	HR = 0.91 (95% CI, 0.83–1.01, NS) intervention group *vs.* control group	[30]
	Meta-analysis (cohort + case-control studies)	RR = 1.091 (95% CI, 1.001–1.184) cohort PUFA RR = 1.042 (95%CI, 1.013–1.073) case-control total fat RR = 1.22 (95% CI, 1.08–1.38) case-control PUFA	[31]
	Systematic review (18 studies)	45–78% increased risk of death with increased intake of *trans* fats	[32]
	EPIC study (337,327 women, 11.5 years follow-up)	HR = 1.20 (95% CI, 1.0–1.45, *p* = 0.05), highest *vs.* lowest quintile of total fat intake (ER^+^PR^+^ BC) HR = 1.2 (95% CI, 1.09–1.52, *p* = 0.009), highest *vs.* lowest quintile of saturated fat intake (ER^+^PR^+^ BC) HR = 1.29 (95% CI, 1.01–1.64, *p* = 0.04), highest *vs.* lowest quintile of saturated fat intake (HER2^−^ BC)	[33]
	Meta-analysis (6 cohort studies + 3 case-control studies)	RR = 1.29 (95% CI, 1.06–1.56), highest *vs.* lowest cholesterol intake	[34]
Dairy products	Pooled analysis (8 prospective cohort studies) (351,041 women, 15 years follow-up)	NS	[35]
	Meta-analysis (18 prospective cohort studies, *n* = 1,063,471)	RR = 0.91 (95% CI, 0.80–1.02, *p* = 0.003), milk consumption RR = 0.85 (95% CI, 0.76–0.95, *p* = 0.01), highest *vs.* lowest total dairy food	[36]
	Meta-analysis (22 cohort + 5 case-control studies)	RR = 0.90 (95% CI, 0.83–0.98, *p* = 0.111), highest *vs.* lowest dairy products RR = 0.91 (95% CI, 0.83–0.99, *p* = 0.991), yogurt consumption RR = 0.85 (95% CI, 0.75–0.96, *p* = 0.121), low-fat dairy consumption	[37]
Carbohydrate, Glycaemic Index	Meta-analysis (19 prospective studies)	RR = 1.04 (95% CI, 1.00–1.07, *p* = 0.19), 10 units/d for glycemic index RR = 1.01 (95% CI, 0.98–1.04, *p* = 0.07), 50 units/d for glycemic load RR = 1.00 (95% CI, 0.96–1.05, *p* = 0.01), 50 g/d for carbohydrate intake	[38]
Soy products, isoflavones	Meta-analysis (14 case-control + 7 cohort studies)	RR = 0.75 (95% CI, 0.59–0.95, *p* = 0.023), soyfood intake RR = 0.81 (95% CI, 0.67–0.99), isoflavone intake	[39]
	Meta-analysis (1 cohort + 7 case-control studies)	OR = 0.71 (95% CI, 0.60–0.85, *p* = 0.023), highest *vs.* lowest soy intake in Asians OR = 0.88 (95% CI, 0.78–0.98, *p* = 0.60), moderate *vs.* lowest soy intake in Asians OR = 1.04 (95% CI, 0.97–1.11, *p* = 0.42), highest *vs.* lowest soy isoflavone intake in Western populations	[40]
	Meta-analysis (18 prospective studies)	RR = 0.89 (95% CI, 0.79–0.99, *p* = 0.001), highest *vs.* lowest isoflavone intake (RR = 0.76, 95% CI: 0.65–0.86, *p* = 0.136 in Asian population; RR = 0.97, 95% CI: 0.87–1.06, *p* = 0.083 in Western population)	[41]

RR: multivariable-adjusted relative risk; HR: adjusted hazard ratio; OR: odds ratio; CI: confidence intervals; NS: not significant; PUFA: poly unsaturated fatty acids; ER: estrogen receptor; PR: progesterone receptor; HER2: human growth factor-neu receptor; BC: breast cancer; EPIC: European Prospective Investigation into Cancer and Nutrition.

**Table 2:** Summary of the evidence (described in Section 5) on nutritional interventions to enhance BC treatment.

	Study	Intervention	Results	Reference
ω-3 PUFAs	Phase II clinical trial (*n* = 25 breast cancer patients, 31 months follow-up)	1.8 g DHA/day anthracycline	Improvement of chemo-therapy outcome: median TTP = 6 months (95% CI, 2.8–8.7 months); median OS = 22 months (95% CI, 17–33 months) No severe adverse side effects (grade 3 or 4 toxicity only for neutropenia and alopecia, 80%)	[99]
	Pilot study (*n* = 38 postmenopausal breast cancer patients)	4 g/day EPA + DHA for 3 months AI therapy	Inhibition of bone resorption in the fish oil responders *vs.* placebo (*p* < 0.05)	[100]
	Controlled clinical trial (*n* = 249 postmenopausal breast cancer patients)	3.3 g/day ω3 PUFA (560 mg EPA + DHA, 40:20 ratio) 24 weeks AI therapy	Reduction of arthralgia (4.36 *vs.* 5.70, *p* = 0.02) obese BC patients *vs.* placebo	[101]
	Controlled clinical trial (*n* = 20 breast cancer patients)	EPA (0.19 g/day) + DHA (1.04 g/day) paclitaxel	Reduction of paclitaxel-induced peripheral neuropathy incidence (OR = 0.3; 95% CI, 0.10–0.88, *p* = 0.029), but not severity (0.95% CI = (−2.06–0.02), *p* = 0.054) EPA + DHA vs. placebo	[102]
Green tea	Prospective cohort study (*n* = 1160 breast cancer patients, 8 years follow-up)	Regular consumption of green tea	Inverse association between regular green tea consumption (≥3 cups/day) and BC recurrence for stage I/II patients (HR = 0.69; 95% CI, 0.47–1.00, *p* < 0.05)	[103]
	Prospective cohort study (*n* = 472 breast cancer patients, 7 years follow-up)	Regular consumption of green tea	Inverse association between regular green tea consumption (≥5 cups/day) and BC recurrence for stage I/II patients (RR = 0.564; 95% CI, 0.350–0.911, *p* < 0.05)	[104]
	Prospective cohort study (*n* = 5042, 9.1 years follow-up)	Regular consumption of green tea	Reduced risk of total mortality (HR = 0.57; 95% CI: 0.34–0.93) and recurrence (HR = 0.54; 95% CI: 0.31–0.96) for the first 60-month post-diagnosis period	[105]
Vitamin C	Controlled clinical trial (*n* = 54 post-menopausal breast cancer patients)	Vitamin C (500 mg) and E (400 mg) +tamoxifen (10 mg twice a day) for 90 days	Decrease of total cholesterol, TG, VLDL (*p* < 0.001) and LDL (*p* < 0.01) *vs.* tamoxifen alone Increase of HDL (*p* < 0.01) *vs.* tamoxifen alone	[106]
	Controlled clinical trial (*n* = 40 breast cancer patients)	Vitamin C (500 mg) and E (400 mg) + 5-fluorouracil (500 mg/m^2^) + doxorubicin (50 mg/m^2^) + cyclophosphamide (500 mg/m^2^) (every 3 weeks for six cycles)	Increase of SOD, CAT, GST, GPx, GSH (*p* < 0.01) *vs.* chemotherapy alone Decrease of MDA, DNA damage (*p* < 0.01) *vs.* chemotherapy alone	[107]
Vitamin E	Prospective cohort study (*n* = 7 breast cancer patients, 30 days follow-up)	Vitamin E (400 mg) + tamoxifen (20 mg daily) for 30 days	Vitamin E supplement interferes with the therapeutic effects of tamoxifen (increase expression of biomarkers of estrogen-stimulation (ER, PR, p-ERK in breast biopsies)	[108]
Vitamin D	Prospective cohort study (*n* = 232 post-menopausal breast cancer patients, 1-year follow-up)	Calcium (1 g) + vitamin D_3_ (800 IU/d and additional 16,000 IU, every 2 weeks) + AI therapy for 1 year	Reduction of AI-associated lumbar spine bone loss: 1.70% (95% CI, 0.4–3.0%; *p* = 0.005) (women with 25(OH)D serum levels ≥40 ng/ml *vs.* women with serum levels <30 ng/ml)	[109]
	Prospective cohort study (*n* = 60 post-menopausal breast cancer patients, 16 weeks follow-up)	50,000 IU/week + AI therapy for 12 weeks	Decrease of disability from joint pain (52 *vs.* 19%; *p* = 0.026); reduction of fatigue (BFI scores 1.4 *vs.* 2.9; NS); reduction of menopausal symptoms (MENQOL scores 2.2 *vs.* 3.2, *p* = 0.035) (women with 25OHD levels > 66 ng/ml *vs.* women with levels < 66 ng/ml)	[110]

AI: aromatase inhibitor; BC: breast cancer; BFI: big five inventory; CAT: catalase; DHA: docosahexaenoic acid; EPA: eicosapentaenoic acid; ER: estrogen receptor; GPx: glutathione peroxidase; GSH: reduced glutathione; GST: glutathione transferase; HDL: high density lipoprotein; HR: hazard ratio; LDL: low density lipoprotein; MDA: malondialdehyde; MENQOL: menopause-specific quality of life; NS: not significant; OS: overall survival; p-ERK: phosphorylated extracellular signal–regulated kinase; PR: progesterone receptor; PUFA: poly unsaturated fatty acids; RR: relative risk; SOD: superoxide dismutase; TG: triglycerides; TTP: time to progression; VLDL: very low density lipoprotein; 25OHD: 25-hydroxycholecalciferol.
